# Quality of life, exercise capacity, cognition, and mental health of Chilean patients after COVID-19: an experience of a multidisciplinary rehabilitation program at a physical and rehabilitation medicine unit

**DOI:** 10.3389/fresc.2023.1274180

**Published:** 2023-12-01

**Authors:** Valeria Paéz, Maria Rodriguez-Fernandez, Diego Morales, Camillo Torres, Andrés Ardiles, Sergio Soza, Cynthia Bustos, Fernanda Manríquez, Cesar García, Rossana Rocco, Morin Lang

**Affiliations:** ^1^ Biomedical Department, Center for Research in Physiology and Medicine of Altitude, Faculty of Health Sciences, University of Antofagasta, Antofagasta, Chile; ^2^ Institute for Biological and Medical Engineering, Schools of Engineering, Medicine and Biological Sciences, Pontificia Universidad Católica de Chile, Santiago, Chile; ^3^ Physical Medicine and Rehabilitation Service, Clinical Hospital of the University of Antofagasta, Antofagasta, Chile; ^4^ Department of Medical Sciences, University of Antofagasta, Antofagasta, Chile; ^5^ Laboratorio de Fisiología del Ejercicio y Metabolismo (LABFEM), Escuela de Kinesiología, Facultad de Medicina, Universidad Finis Terrae, Santiago, Chile; ^6^ Departamento de Ciencias de la Rehabilitación y Movimiento Humano, Facultad Ciencias de la Salud, Universidad de Antofagasta, Antofagasta, Chile

**Keywords:** coronavirus, SARS-CoV-2, post-COVID disabilities, rehabilitation, multidisciplinary team

## Abstract

**Background:**

Post-COVID disabilities, encompassing physical, cognitive, and psychological aspects, constitute the primary health sequelae for survivors. While the rehabilitation needs post COVID-19 are now well understood, each country possesses unique characteristics in terms of populations, healthcare systems, social dynamics, and economic profiles, necessitating context-specific recommendations. This study aims to address two main objectives: (1) analyze the impact of an 8-week multidisciplinary rehabilitation program on the quality of life, functional capacity, cognition, and mental health adaptations in adults recovering from COVID-19 in northern Chile, and (2) propose a personalized model for predicting program dropouts and responses.

**Methods:**

A total of 44 subjects were enrolled, forming two groups during the study: a treatment group (*n* = 32) and a dropout group (*n* = 12). The treatment group participated in the 8-week multidisciplinary rehabilitation program.

**Results:**

The results indicate that (1) After 8 weeks, the quality of life of the patients in the treatment group exhibited significant improvements reflected in all aspects of the Short Form-36 Health Survey (SF36, *p* < 0.005) and the total score (*p* < 0.001), with a concurrent decrease in dysfunctionality (*p* < 0.001). (2) Significant improvements were also observed in various physical performance tests, including the: 6-minute walk test, 1-min sit-to-stand, dynamometry, Tinetti balance, and Berg score (*p* < 0.001). Moreover, physical therapy led to a reduction in neuropathic symptoms and pain, psychological therapy reduced anxiety and depression, and language therapy enhanced memory and speech (all *p* < 0.05). (3) Demographic and clinical history characteristics did not predict responses to rehabilitation. (4) A regression model for predicting changes in SF-36 total score, based on physical function, physical role, general health, and mental health, was established based on the data from study (*p* < 0.01, adjusted *R*^2^ = 0.893). (5) Classification models for predicting dropouts achieved 68% accuracy, with key predictors of treatment adherence including diabetes, hypertension, and dyslipidemia, Tinetti balance, physical role, and vitality of SF36, and performance on the 6-minute walk test and 1-minute sit-to-stand.

**Conclusions:**

This study demonstrates significant enhancements in quality of life, improved functional performance, and reductions in mental and cognitive burdens within an 8-week rehabilitation program. Additionally, it is possible to identify patients at risk of dropping out using cost-effective, outpatient, and clinically applicable tests.

## 1. Introduction

Severe acute respiratory syndrome coronavirus-2 (SARS-CoV-2), the virus responsible for COVID-19, first emerged in December 2019 in Wuhan, Province of Hubei, China, and rapidly spread globally, eventually leading to its declaration as a pandemic by the World Health Organization (WHO) in March 2020 (1). In Chile, the peak of infections occurred on June 16, 2021, with 3.301 individuals requiring intensive care unit (ICU) hospitalization. Among them, 37.17% were older adults (≥60 years), and a staggering 82.26% did not survive (2).

COVID-19 presents significant challenges, both in terms of its immediate health impact and its long term health consequences. Among the foremost health outcomes associated with Post Intensive Care Syndrome (PICS) in COVID-19 survivors are physical, cognitive, and psychological sequelae (3–5). PICS, as defined by Vrettou et al. (6), encompasses “new or worsening impairments in physical, cognitive, or mental health status arising after critical illness and persisting beyond acute care hospitalization”. The risk of developing PICS is particularly associated with severe clinical conditions, such as acute respiratory distress syndrome (ARDS) (6, 7), which are often observed in COVID-19 patients. The emergence of post-COVID-19-PICS presents a substantial challenge to public health (6), predominantly affecting COVID-19 survivors who face an elevated risk of PICS compared to other critically ill (6, 8). The symptoms encompass psychological conditions like anxiety and depression, physical dysfunction including breathlessness, weakness, fatigue, chronic pain, and cognitive impairments, manifesting as issues in memory, attention, and speed of mental processing (6, 7).

On the other hand, the continuation or development of new symptoms occurring 3 months after the initial infection is referred to as “long COVID”. While the symptoms of this condition can vary and impact multiple body systems, recent reviews have highlighted common manifestations, including fatigue, cognitive dysfunction, and respiratory symptoms (9, 10).

The impairment of health and functional performance in individuals who experienced moderate to severe cases of COVID-19, has underscored the importance of promoting rehabilitation to restore quality of life and optimal functionality (11–13). Various authors have studied treatment protocols and recommend comprehensive rehabilitation approaches for long-term COVID-19 illness, including exercise, nutrition, education, voice control, breathlessness management, neurocognitive interventions, mental health support, addressing eating difficulties, and assistance with daily activities (4, 14, 15). To effectively restore the health of these individuals and ensure long-term well-being, the promotion of multidisciplinary rehabilitation teams and integrated management is essential (4, 8, 14, 15).

Despite the understanding of rehabilitation needs after covid-19 and its impact on the population, each country has unique population characteristics, health systems, social dynamics, and economic profiles. Furthermore, these characteristics can vary across different regions within a country (16), necessitating studies that provide context-specific recommendations.

When prescribing multidisciplinary treatment to post-hospitalized patients, swift action is recommended to restore physical capacities, and motor skills, work abilities, social functioning, and emotional well-being (4). However, not all patients readily accept this type of therapy, leading to treatment discontinuation, as observed in previous studies on COVID-19 of (8), and missed opportunities within the healthcare system due to non-adherence to multidisciplinary treatment for other conditions (17–19). Therefore, there is a clinical need for models or scores that can predict, with a sufficient degree of confidence, which patients are more likely to complete a rehabilitation program. This prediction is crucial, particularly in clinical context, with limited resources, especially when public funds support the participants, as was the case in our study.

This study was conducted a university hospital in northern Chile, with participants benefiting from the public health system, and aimed to contribute to the community without, cost to the participants. This study aimed to achieve two primary objectives: first, to analyze the impact of a multidisciplinary rehabilitation program on the quality of life, exercise capacity, cognition, and mental health of post-COVID-19 adults in northern Chile, and second, to propose a personalized model for predicting dropouts from such a rehabilitation program.

## 2. Methods

### 2.1. Subjects

**Figure 1:**
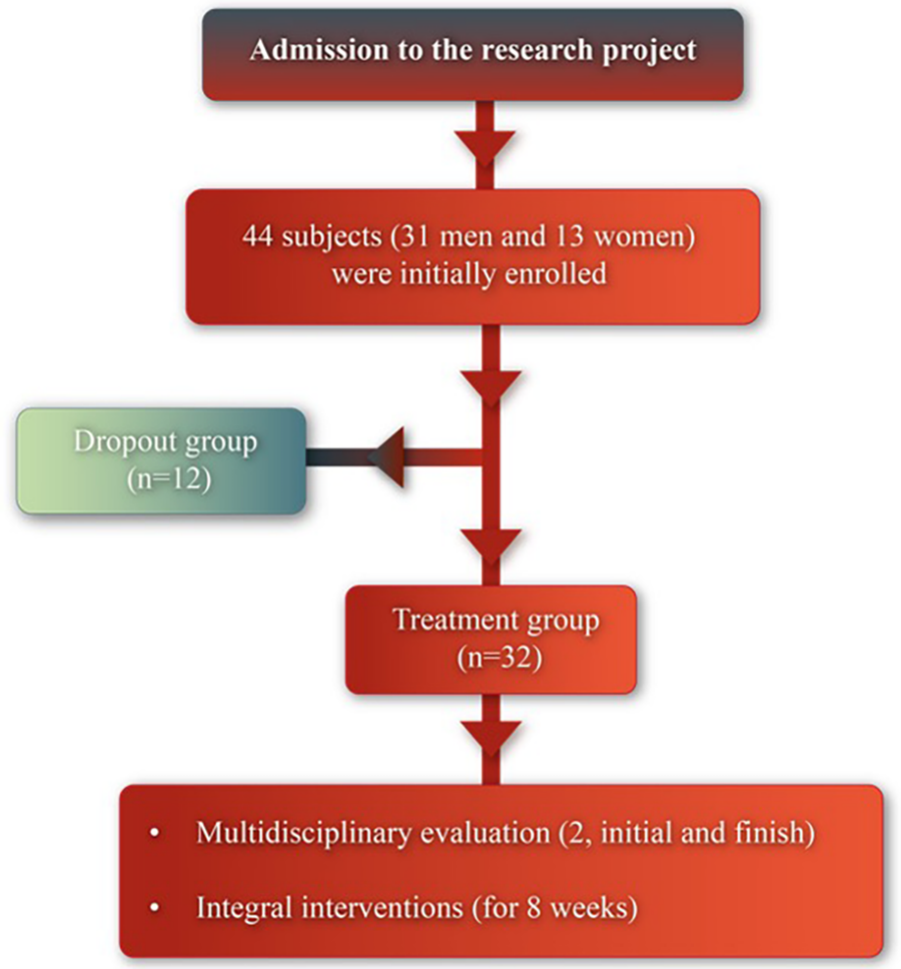
Flow diagram of the study.

A total of 44 subjects (31 men and 13 women) were initially enrolled in this non-randomized experimental prospective study. Two groups were formed throughout study: the treatment group (30 subjects, 56.03 ± 14.05 years old) and the dropout group (12 subjects, 56.33 ± 11.33 years old), which consisted of subjects who dropped out of the program (see Figure 1).

To be eligible for the study, participants had to meet the following criteria: moderate presentation of COVID-19 illness (patients hospitalized in intermediate care units without ICU criteria) or severe presentation of COVID-19 illness (patients requiring ventilatory support in intensive care); elapsed time from hospital discharge less than 1.5 years; age above 18 years; adequate level of comprehension (Mini Mental State Examination, MMSE >26 points); chronic pathologies under pharmacological treatment and control; literacy; absence of suicidal ideation (according to C-SSRS Baseline-Screening); seeking rehabilitation services for the first time; and signed informed consent in writing for participation in the study. Participants with pre-existing cardiovascular, metabolic, and respiratory diseases were evaluated by the medical teams to ensure secure control and stabilization.

The study obtained approval from the Ethics Committee in Scientific Research of the University of Antofagasta (CEIC-UA) (approval #339/2021) and complies with the standards specified in the Nuremberg Code, Declaration of Helsinki, CIOMS, and guidelines of Ezekiel Emanuel. The study was conducted at the Physical Medicine and Rehabilitation Unit of the Clinical Hospital of the University of Antofagasta, Chile, and involved personnel from the University of Antofagasta. The University of Antofagasta Clinical Hospital, established in 2019 through a collaboration between the University of Antofagasta and the Antofagasta Regional Government, serves the dual purpose of advancing specialist training, addressing the scarcity of such professionals in the region, and creating a clinical environment for educating medical healthcare sciences students across diverse specialties. Furthermore, it offers training opportunities for professionals in fields like Medical Technology, Nursing, Obstetrics, Kinesiology, and Occupational Therapy.

### 2.2. Experimental procedure

A call was made to the Antofagasta community through the social networks of the Clinical Hospital of the University of Antofagasta, as well as through television and radio in the city.

**Figure 2:**
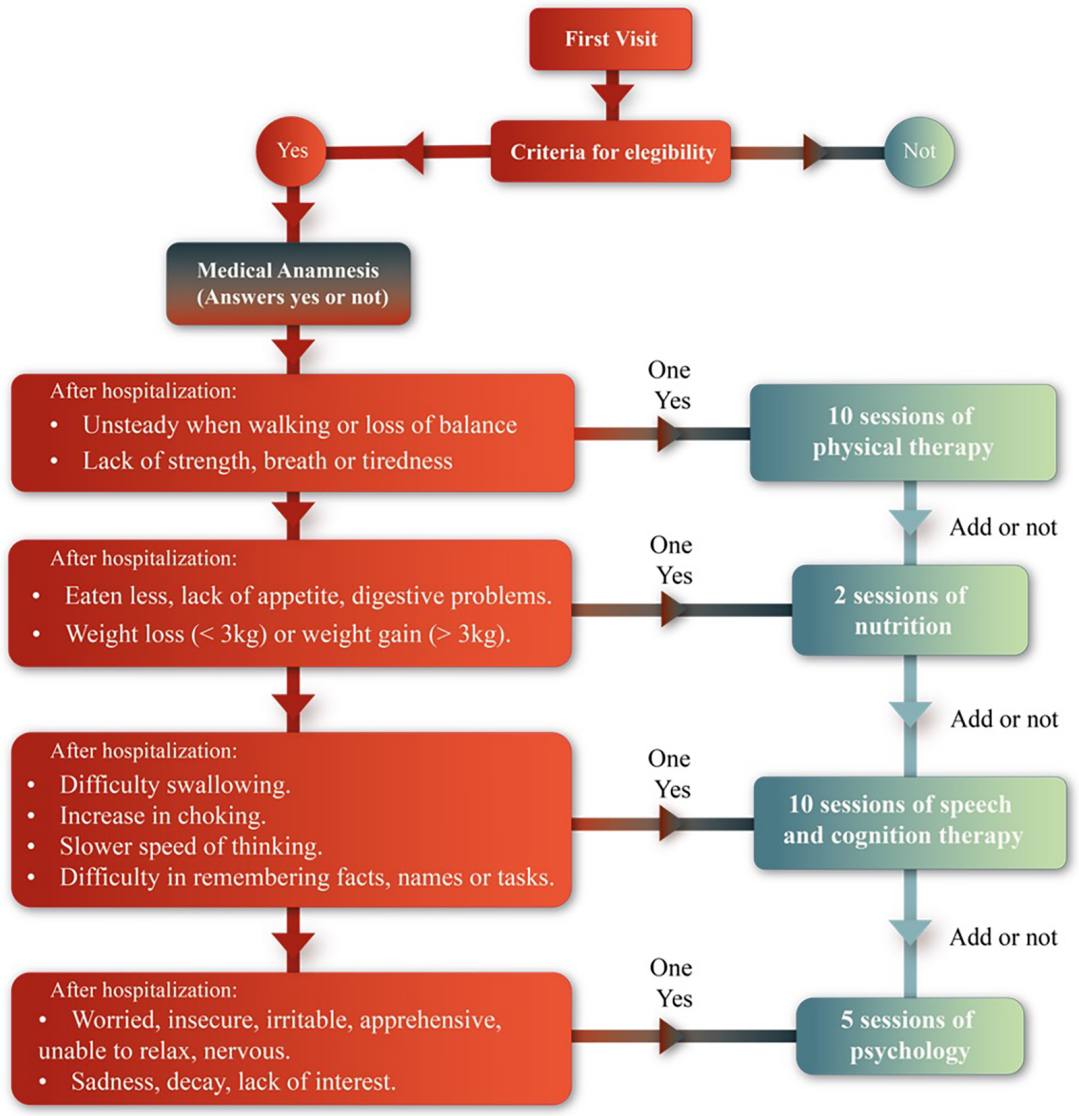
Medical anamnesis.

Participants who met the eligibility criteria entered the study after a medical anamnesis and pharmacological assessment to determine the appropriate rehabilitation for their needs (see Figure 2).

**Figure 3:**
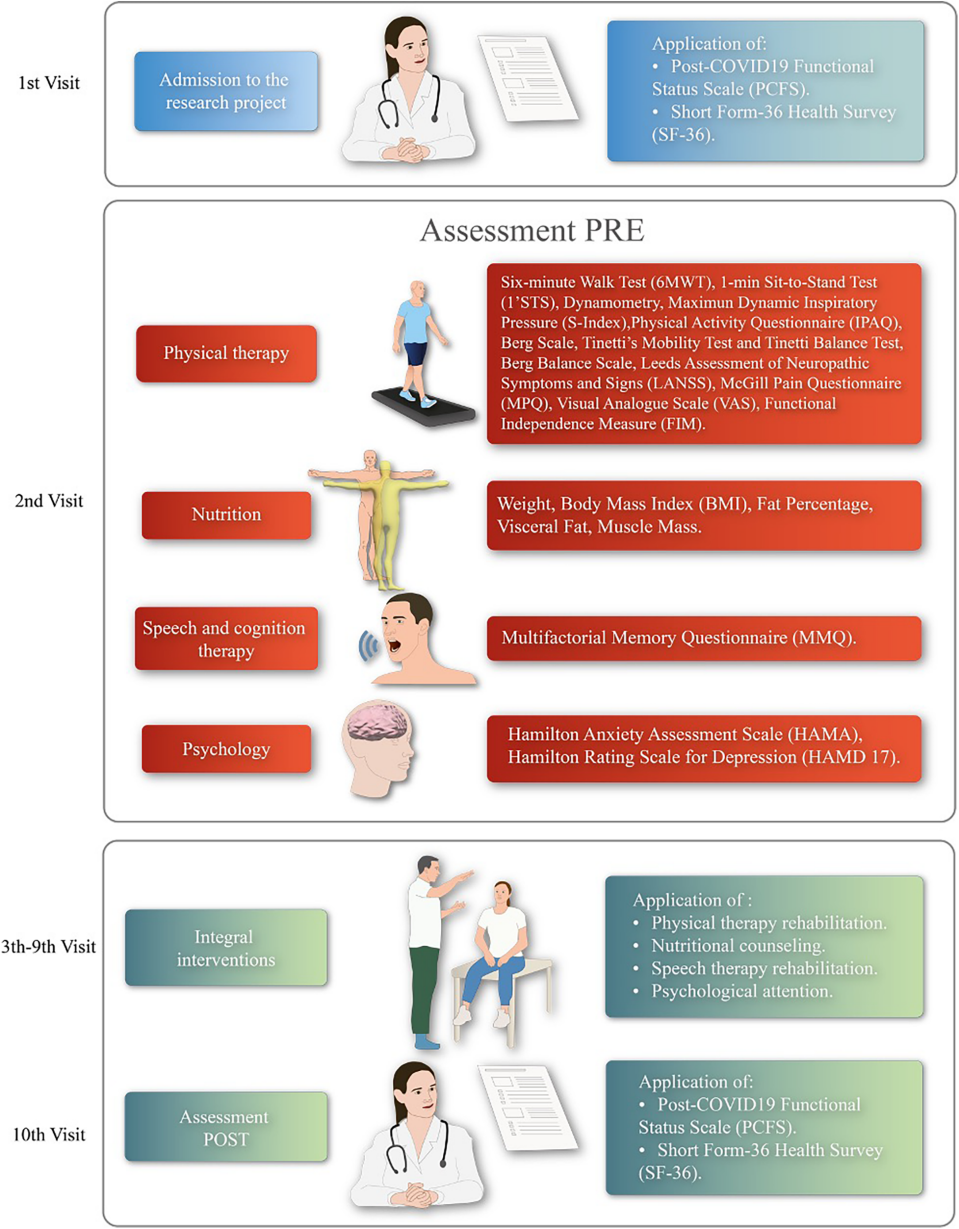
Experimental protocol overview. PRE, baseline evaluations of each discipline; POST, evaluations after 8 weeks of interventions.

The participants underwent an 8-week treatment program with 100% attendance compliance. The treatment included: physical therapy twice a week, speech and language therapy twice a week, psychological therapy once a week, and nutritional consultation once a month (see Figure 3).

The order of administration assessments was randomized, and participants were instructed to request rest breaks as needed. Trained investigators from the Physical Medicine and Rehabilitation Service of HCUA, together with last-year university students in practice at the University of Antofagasta, conducted data collection.

Vital signs of the patients were monitored by medical staff and students during clinical assessments and therapies.

We followed the TIDieR (Template for Intervention Description and Replication) checklist to provide a comprehensive and standardized description of our rehabilitation intervention (20).

### 2.3. Quality of life and functionality assessment

The quality of life was assessed using the Spanish version of the Short Form-36 Health Survey (SF-36) questionnaire (21), which is used in the Public Health System in Chile (22). The questionnaire consists of 36 items that cover various scales related to: physical function, physical role, body pain, general health, vitality, social function, emotional role, and mental health. The questionnaire was self-administered by the patients in a quiet room of the hospital following the methodology described by Vilagut et al. (21). To consider a scale as valid, patients were required to have answered at least 50% of its items. To calculate the score, any unanswered item was substituted with the average value derived from the completed items.

Functionality was estimated using the post-COVID-19 Functional Status (PCFS) Scale (23), a Spanish version recommended by Scientific Societies and Professional Colleges of the Rehabilitation Area (15). The scale was applied through a structured interview conducted by trained researchers at the beginning and end of the 8-weeks rehabilitation program.

### 2.4. Multidisciplinary assessment

#### 2.4.1. Cardiorespiratory assessment

The cardiorespiratory assessment included the following tests:

• The 6-minutes walking test (6MWT) was conducted following the American Thoracic Society protocol (24). Vital signs were measured using an oscillometer device (AND UA-767Plus, Japan), and a pulse oximeter (Vantage 9590, NONIN, USA) before starting the test, at the end of the rest, and 5 min post-recovery. The total distance covered in meters (m) and the percentage predicted distance reached for Chilean individuals, in agreement with reference values (25), were calculated.
• Muscular strength and endurance of the lower body were measured using the 1-min sit-to-stand test (1'STS), recommended for post COVID-19 patients in Chile (26). This test followed the methodology described by Strassmann et al. (27), using a chair (height 46 cm) without arm rests. The results were compared with reference values (27).
• Muscular strength and endurance of the upper body was measured using hand grip test according to the standardized protocol described in (28) for adult Chileans.
• Maximum dynamic inspiratory pressure (S-Index) was measured using the K5, POWER-Breathe® Series K device. The device was kept clean using cleaning pills for Powerbreathe and disposable filters (29). The measurement process involved a warm-up consisting of a series of 30 breaths with a pressure load at 40% of the best S-Index measured in the first 3 maneuvers before the start of the warmup. Participants were encouraged to make a maximum effort until reaching total pulmonary capacity with vigorous verbal stimulation. Immediately afterward, the test was performed exhaling softly but completely, followed by a forceful, fast, and deep inhalation until the lungs were completely full. At least 8 consecutive maneuvers were performed at a rhythm of between inspirations, with 60 s of rest between maneuvers (30).
• Physical activity level was measured per week using the International Physical Activity Questionnaire (IPAQ) (31).

All participants were familiarized with the test procedures before evaluation. The assessments were conducted during the first session and the final session (10th) after 8 therapy sessions.

#### 2.4.2. Motor assessment

Motor assessment included Tinetti's Mobility Test and Tinetti Balance Test, based on the methodology described by Tinetti et al. (32). The Berg Balance Scale was also employed to assess balance according to Berg et al. (33). Each subject was evaluated during the first session and the final session (10th) after 8 therapy sessions. Scores ranging from 0 to the maximum score for each category were assigned to rate the performance of patients in each physical task. The scores were summed (/56 for Berg balance scale, /16 for Tinetti's Mobility Test, and /12 for Tinetti Balance Test).

#### 2.4.3. Pain assessment

Pain assessment involved the following evaluations:

• The presence of neuropathic pain, defined as pain resulting from a nerve dysfunction or pathological change, was determined using the Leeds Assessment of Neuropathic Symptoms and Signs (LANSS) questionnaire based on Bennet et al. (34). The assessment was conducted through an interview, where patients described the nature of the pain that they experienced the previous week and a physical examination to assess for allodynia and pain threshold.
• The McGill Pain Questionnaire (MPQ) (35) was used to obtain pain description (PRI) based on the patients’ chosen words to describe their pain from 20 subgroups, as well as pain intensity (PPI) rated on a scale of Light (1), Annoying (2), Distressing (3), Horrible (4) and Atrocious (5). Additionally, pain intensity at the time of the interview was evaluated using a 100 mm visual analogue scale (VAS). The assessments were conducted during the first session and the final session (10th) after 8 therapy sessions.

#### 2.4.4. Nutritional assessment

Body composition was assessed using a 5-segment electrical bioimpedance device (InBody 120, Japan), and software (LookinBody 120, Japan) following practical guidelines outlined by Walter-Kroker et al. (36), and the Inbody 120 manual (37). Participants were measured while fasting, standing for 5 min before the test. Participants were instructed not to engage in physical exercise 24 h prior to the test and to empty their bladder and bowels. The assessments were conducted at the beginning of each month on 2 occasions.

#### 2.4.5. Occupational therapy assessment

Functional evaluation of activities of daily living was performed using the Functional Independence Measure (FIM), according to Paolinelli et al. (38). Scores ranging from 0 unable to perform to maximum score of 120 were assigned to rate the performance of patients in each requested physical task. The FIM score was obtained during the first session and the final session (10th) after 8 therapy sessions, respectively.

#### 2.4.6. Speech and cognition therapy assessment

Cognition assessment was conducted using the Multifactorial Memory Questionnaire (MMQ), according to (39). The subject's cognition was classified as: below average (30–39), average (40–60), or above average (60–70) based on the obtained t-score. The assessments were performed during the first session and the final session (10th) after 8 therapy sessions.

#### 2.4.7. Psychological assessment

Psychological assessment involved the following evaluations:

• Depression assessment was carried out using the Hamilton Rating Scale for Depression (HAMD-17), based on Pistarini et al. (40). Participants were classified into different categories: no depression (0–7 pts), mild depression (8–12 pts), moderate depression (13–17 pts), severe depression (18–24 pts), and very severe depression (30–52 pts).
• Anxiety was assessed using the Hamilton Anxiety Assessment Scale (HAMA), according to Hamilton (41) and Chadli (42). Participants were classified into different categories: no anxiety (0 pts), mild anxiety (<17 pts), moderate anxiety (18–24 pts), and severe anxiety (25–30 pts).

Both scales were applied during the first session and the final session (5th) after 4 therapy sessions.

### 2.5. Intervention protocols

#### 2.5.1 Cardiorespiratory interval protocol

The intervention was administered by an academic physiotherapist from the University of Antofagasta, holding a post-degree in rehabilitation. The treatment was conducted in-person through individual sessions, occurring three times a week.

The cardiorespiratory intervention consisted of aerobic interval training (AIT) using treadmill or cycle ergometer exercises. The protocol included 6 min of exercise followed by 2 min of rest, repeated for 30 min. Workload interval intensities were defined as moderate (60%–70% of HR reserve (HRR) and rating of perceived exertion (RPE) between 5 and 6 on the Borg Category Ratio Scale anchored at number 10 (Borg CR-10), and recovery interval intensity as low (greater than 40% of HRR and RPE between 2 and 3 on the Borg CR-10 scale). During the aerobic training, oxygen saturation (SpO_2_) was monitored to maintain levels above 90%, and supplemental oxygen was administrated when necessary. The muscular strength protocol (SP) included 3 functional muscular exercises (Supplementary Figure S1), with repetitions performed for 30 s followed by a 15-second rest, repeated 4 times. The exercise intensity progressed from moderate (5–6 on the Borg CR-10 scale) to high (7–8 on the Borg CR-10 scale), at workload between 50% and 60% of one maximum repetition, for a total duration of 15 min. Active respiratory exercises were applied: diaphragmatic breathing, supported coughing, chest stretching with hands above the head, in addition to the use of an air flow increaser (600–900–1,200/Triflow). The treatment was based on guidelines for “Managing breathlessness” and “Physical activity and Exercise from Moderate-intensity activity” by WHO (43) and Kinesiology Recommendations by Scientific Societies and Professional Associations of the rehabilitation area of Chile (15).

#### 2.5.2. Motor protocol

The intervention was administered by an academic physiotherapist from the University of Antofagasta, holding a post-degree in rehabilitation. The treatment was conducted in-person through individual sessions, occurring three times a week.

The motor intervention protocol included muscle strengthening exercises, proprioceptive exercises, balance training, gait re-education. The treatment protocol was based on guidelines provided by Tinetti et al. (44) and “Kinesiology Recommendations” by Scientific Societies and Professional Associations of the rehabilitation area of Chile (15). The standardized guideline of exercises is shown in Supplementary Figure S2.

#### 2.5.3. Pain protocol

The intervention was administered by a physiotherapist from the Clinical Hospital of the University of Antofagasta, holding a post-degree in rehabilitation. The treatment was conducted in-person through individual sessions, occurring three times a week.

The pain intervention protocol included pain pathophysiology education, pain management education, and the application of non-invasive soft tissue management techniques. The treatment was based on guidelines outlined in: “Managing Pain” by WHO (43) and “The Management of Pain in the Process of Chronification” by Scientific Societies and Professional Associations of the rehabilitation area of Chile (15). The standardized guideline of exercises is shown in Supplementary Figure S3.

#### 2.5.4. Nutritional protocol

The intervention was administered by an academic nutritionist from the University of Antofagasta, holding a post-degree in rehabilitation. The treatment was conducted in-person through individual sessions, occurring once a month.

The nutritional intervention protocol involved providing food guidelines with caloric adequacy based on the results of body composition assessments and underlying diseases. Additionally, counseling and education were provided. The calculation of dietary intake adequacy was based on guidelines provided in: “Nutrition and eating a healthy and balanced diet” by WHO (43), “Nutritional considerations to favor the rehabilitation process” by Scientific Societies and Professional Colleges of the rehabilitation area of Chile (15), and “Nutritional recommendations for caring for infected people with COVID-19” by Nazarena et al. (45).

#### 2.5.5. Occupational therapy protocol

The intervention was administered by an academic occupational therapist from the University of Antofagasta, holding a post-degree in rehabilitation. The treatment was conducted in-person through individual sessions, occurring three times a week.

The occupational therapy intervention aimed to educate patients on joint protection techniques, increase upper limb strength, and enhance grasping skills. The treatment protocol followed the recommendations outlined in the “Recommendations for Intensive Occupational Therapy” by Scientific Societies and Professional Associations of the rehabilitation area of Chile. The standardized guideline of exercises can be found in Supplementary Figure S4.

#### 2.5.6. Cognition and deglutition protocol

The intervention was administered by an academic speech-language pathologist from the University of Antofagasta, holding a post-degree in rehabilitation. The treatment was conducted in-person through individual sessions, occurring three times a week.

The speech intervention focused on implementing adaptation strategies in the environment, conducting rehabilitation exercises, teaching postural techniques, and practicing swallowing maneuvers. The treatment protocol was based on the guidelines provided in: “Managing problems with attention, memory, and thinking clearly” by WHO (43) and “Speech Therapy Recommendations” by Scientific Societies and Professional Associations of the rehabilitation area in Chile (15).

#### 2.5.7. Psychological protocol

The intervention was administered by a psychologist from the Clinical Hospital of the University of Antofagasta, holding a post-degree in rehabilitation. The treatment was conducted in-person through individual sessions, occurring at least once a week.

The psychological intervention involved relaxation exercises, promoting rewarding activities, cognitive restructuring, enhancing self-esteem, and fostering resilience. The treatment protocol was based on the guidelines presented in: “Man aging stress, anxiety, depression and sleep problems” by WHO (43).

### 2.6. Statistical analysis

The sample size was calculated using the software G*Power 3.1, considering the Wilcoxon test for two related samples to analyze the changes between baseline and 8 weeks of intervention. A bilateral contrast, an effect size of *d* = 0.5, a significance level of *α* = 0.05, and a power of 0.80 resulted in a minimum sample size of 34. To account for potential variables with smaller effect sizes, we increased the sample size to 44.

All statistical analyses were performed using MATLAB (MATLAB_R2022a, 2022). The significance level was set at *α* = 0.05 (two-sided) for all tests. Data are reported as means and standard errors [mean (SD)] or as numbers and percentages [*n* (%)].

The Shapiro-Wilk test was used to assess the data distribution, and most of the data was found to follow a normal distribution. For normally distributed data, the differences between baseline and 8 weeks of interventions were evaluated using the paired Student's *t*-test. Non-normally distributed variables were analyzed, using the non-parametric Wilconxon sign-rank test for paired samples and Pearson chi-square (*X*^2^) for proportions. In cases where two variables had different distributions, the non-parametric test was chosen. The effect size of the differences between groups and conditions was computed as Cohen's-*d* by Ruggero G. Bettinardi (46).

Logistic regression models were employed to predict the responder type: good or poor. A good responder was defined as an individual who experienced an increase equal to or greater than 30% in the delta of the SF-36 total score, which was calculated using the formula [(end—start)/end] * 100. The predictors considered in the analysis were age, sex, COVID-19 category, length of hospital stay, time elapsed after hospital discharge, pre-hospitalization vaccinations, post-hospitalization vaccinations, arterial hypertension (HT), diabetes mellitus (DM), dyslipidemia, depression, respiratory disease (asthma or chronic obstructive pulmonary disease), smoking status, and alcohol consumption.

The delta of SF-36 total score for patients who underwent rehabilitation was predicted using a multivariate linear regression model with backward selection. This approach initially included all variables in the model and subsequently removed the least significant ones (*p* ≥ 0.05) until all remaining variables were significant in explaining the outcome. The predictors considered in the analysis were the deltas of changes in the nine areas assessed by the SF-36 questionnaire, which encompassed physical function, physical role, body pain, general health, vitality, social role, emotional role, mental health, and health transition.

Finally, the Classification Learner App available in the Matlab Statistics and Machine Learning Toolbox (Mathworks, Natick MA), was employed to develop a classification model able to predict dropouts. A comprehensive set of 86 predictors was considered, including general data variables (such as age, sex, days of hospitalization, among others) and baseline variables (pre-intervention outcomes). The Chi2 algorithm for feature ranking (47) was employed to select the top 10 highest-ranking features.

Several classifiers, including Coarse tree, Medium Tree, and Fine Tree, were evaluated, with Coarse tree yielding the best results. The Coarse tree classifier was trained using different values for the cost matrix, enabling the assignment of different misclassification costs to each type of error. This approach is particularly crucial in imbalanced datasets, such as the one in this study, where the number of subjects in the treatment group is almost three time higher than the number of subjects in the dropout groups (positive group) Therefore, false negatives (FN) were penalized with a cost of 3, and false positives (FP) were penalized with a cost of 1. This penalty scheme aimed to optimize the model's performance in classifying the minority class. Furthermore, 5-fold cross-validation was performed to assess the model's performance. The model underwent 100 iterations, each trained using the fitctree function and the top 10 highest-ranking features determined by fscchi2. The accuracy of these 100 iterations was calculated, and the mean value, along with the standard deviation (SD) was reported. It is worth noting that the fitctree function excludes observations with entirely missing values for the predictor vector. However, observations with some missing values are used to find splits on variables where those observations have valid values (48).

## 3. Results

A total of 44 Post-COVID-19 survivors enrolled in the program were included in the analysis, with 32 completing the 8-week interventions. Twelve patients discontinued the program before 8 weeks due to various reasons, such as repeated absences, lack of motivation, loss of telephone follow-up, among others. Among the 44 patients, 29 were treated in the ICU and required ventilatory support, while 15 were treated in intermediate or medium care units without ICU hospitalization criteria.

**Figure 4:**
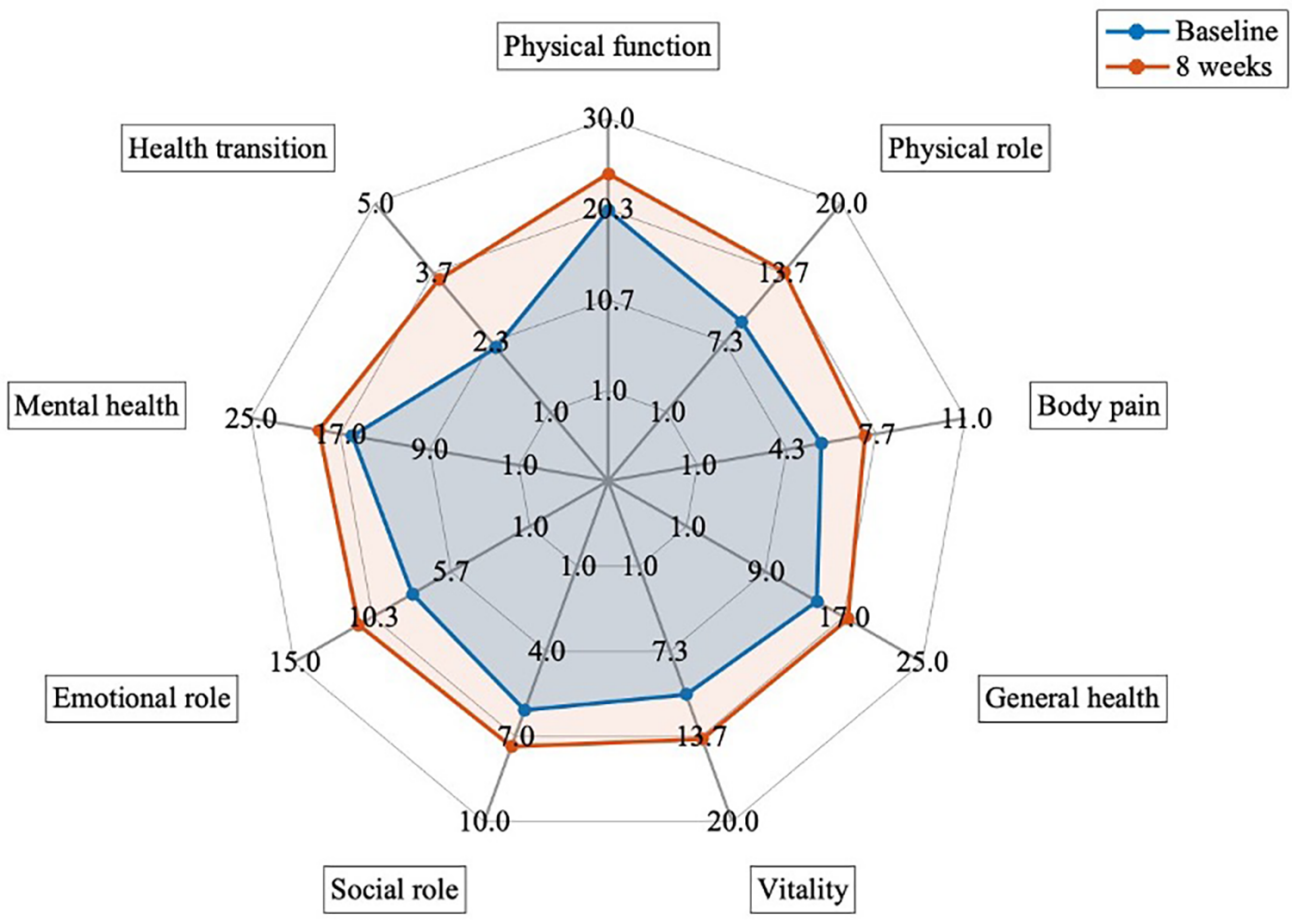
Comparison of quality of life measured by SF36 questionary between baseline and after 8 weeks of interventions (*n* = 32). *p*-value <0.005 for all variables.

**Figure 5:**
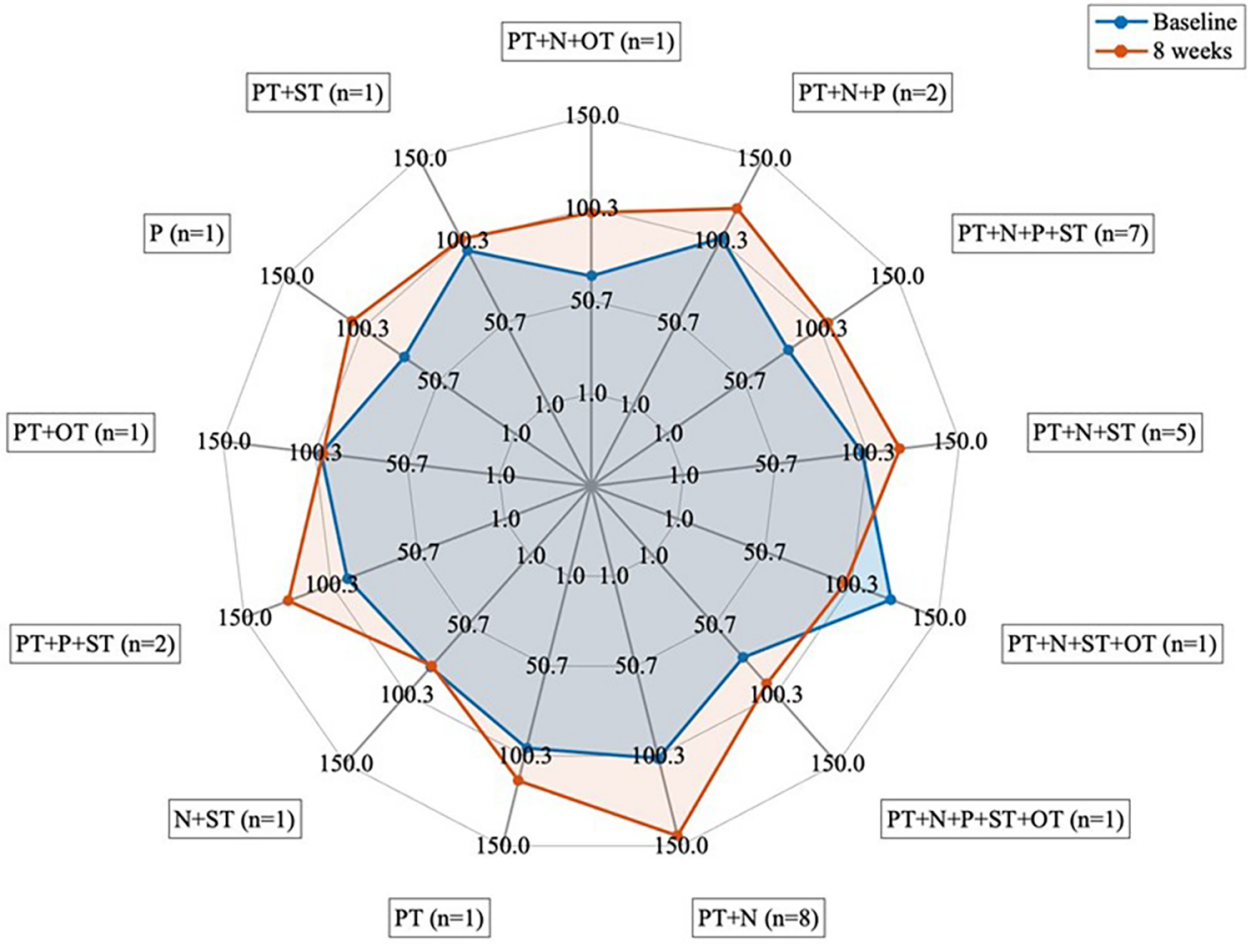
Groups based on the type of therapy received (*n* = 32). PT, physical therapy; N, nutrition; OT, occupational therapy; P, psychology; ST, speech therapy.

Baseline characteristics of the patients are presented in Table 1. The quality of life significantly improved after the 8-week program (*p*-value <0.001, *d* = −1.23, Figure 4, and Supplementary Table S1). The level of dysfunctionality, as measured by PCFS, also showed a significant reduction after 8 weeks of intervention (Table 1, *p*-value <0.001, *d* = 0.689, Supplementary Table S2). The 32 patients received a multidisciplinary intervention tailored to their individual requirements, as evaluated during the medical anamnesis (Figure 5 and Supplementary Table S3).

**Table 1:** Patient characteristics at the beginning and end of the outpatient COVID-19 rehabilitation program.

1. Demographics and history		
	Moderate covid	Severe covid
Number	15	29
Age, years^+^	62.53 (12.42)	52.79 (12.59)
Male, *n*	11 (73.3%)	20 (68.96%)
History of HT, *n*	10 (66.6%)	16 (55.17%)
History of DM, *n*	4 (26.6%)	6 (20.68%)
History of Dyslipiemia, *n*	3 (20%)	10 (34.48%)
History of depression^+^	1 (66.6%)	10 (34.48%)
History of respiratory disease (Asthma or COPD), *n*	3 (20%)	9 (31.03%)
Current or Ex-smoker, *n*	1 (6.66%)	2 (6.89%)
Alcohol, *n*	7 (46.6%)	9 (31.03%)
Length of hospital stay, days^+++^	17.93 (30.47)	54.89 (43.34)
Required ICU admission, *n*	0 (0%)	29 (100%)
Pre-hospitalization vaccinations, *n*	3 (20%)	1 (3.44%)
Post-hospitalization vaccinations, *n*	12 (80%)	26 (89.65%)
Time after hospital discharge, days	2.13 (0.91)	2.27 (0.84)
2. Quality of life and functionality		
	Baseline	8 weeks
Number	44	32
Total score of SF36, pts^+++^	92.03 (22.09)	117.34 (26.59)
Dysfunctionality by PCFS^A^, level^+++^	3.03 (0.96)	2.40 (1.29)
Dysfunctionality by PCFS ≥3, *n*	27 (84.37%)	17 (53.12%)
3. Physical assessment		
	Baseline	8 weeks
Cardiorespiratory assessment		
Number	24	24
6MWD, m^+++^	483.29 (142.95)	527.79 (137.59)
6MWD, % of predicted^+++^	80.10 (23.57)	90.24 (19.36)
1'STS, rep^+++^	25 (11.81)	36 (13.42)
S-index ^B*++^	76.82 (25.73)	87.72 (24.41)
Dynamometry, kg^++*^	28.29 (13.02)	33.29 (13.95)
Mets/week^C++^	332.26 (393.91)	742.89 (513.52)
Motor assessment		
Number	15	15
Tinetti balance, pts^+++^	11.93 (4.44)	14.80 (1.82)
Tinetti march, pts^+^	9.33 (3.82)	10.53 (2.99)
Tinetti score, pts^+++^	21.26 (7.75)	25.26 (4.49)
Berg score, pts^+++^	40.60 (15.18)	49.46 (9.53)
Pain assessment		
Number	6	6
Lanss scale, pts^+^	17.33 (5.24)	8.16 (4.75)
Pain intensity (PPI) by McGill questionnaire^D+^	3.16 (1.16)	1.66 (0.51)
Description and pain assessment index (PRI) by Mc-Gill questionnaire^+^	36.66 (15.90)	15.33 (8.26)
EVA, pts^+^	8.83 (0.98)	3.66 (1.36)
Nutritional assessment		
Number	24	24
Weight, kg	86.60 (17.89)	86.84 (18.80)
IMC, kg/m^2^	32.40 (6.81)	32.31 (6.68)
Fat, %	37.19 (9.96)	38.33 (9.32)
Visceral fat, level	16.75 (7.16)	16.62 (7.25)
Muscular mass, %	34.11 (5.86)	35.61 (10.15)
Occupational therapy assessment		
Number	3	3
FIM score, pts	89.33 (33.23)	106.66 (6.11)
4. Mental burden		
	Baseline	8 weeks
Number	13	13
HAMA, anxiety level^E++^	2.07 (0.64)	0.53 (0.77)
HAMA, anxiety level ≥2, *n*	11 (84.61%)	2 (15.38%)
HAMD, depression level^F++^	2.15 (0.98)	0.23 (0.59)
HAMD, depression level ≥2, *n*	11 (84.61%)	1 (7.69%)
5. Cognitive burden		
	Baseline	8 weeks
Number	18	18
Capacity score by MMQ, pts^+^	44.77 (7.2)	49.83 (9.89)
Capacity level by MMQ <2^G^, *n*	3 (16.66%)	3 (16.66%)
Ability score by MMQ, pts^++^	44.66 (10.23)	52.77 (10.78)
Ability level by MMQ <2^G^, *n*	8 (44.44%)	2 (11.11%)
Strategy score by MMQ, pts	41.38 (7.78)	41.77 (7.04)
Strategy level by MMQ <2^G^, *n*	8 (44.44%)	6 (33.33%)

Data are mean (SD) and *n* (%), where *n* is the total number of participants with available data. COPD, chronic obstructive pulmonary disease, pts, points, +, *p* < 0.05, ++, *p* < 0.005, +++, *p* < 0.001. (A) Dysfunctionality levels (PCFS): 0 = No functional limitations, 2 = No significant functional limitation, 3 = Moderate functional limitation, 4 = Severe functional limitation, F, deceased. (B) S-index (POWERbreathe K5): *n = 22 (2 participants did not show up for their final evaluations). (C) Exercise frequency (meets/week, IPAQ). (D) Pain intensity (Mc-Gill questionnaire): 1 = mSlight, 2 = snnoying, 3 = snxious, 4 = horrible and 5 = atrocious. (E) Anxiety levels (HAMA): 0 = no anxiety, 1 = mild anxiety, 2 = moderate anxiety, 3 = severe anxiety. (F) Depression level (HAMD): 0 = not depressed, 1 = mild depression, 2 = moderate depression, 3 = severe depression, 4 = very severe depression. G. MMQ levels: 1 = below average, 2 = average, 3 = above average.

Most physical variables showed significant improvements after the 8-week program, as indicated in Table 1. However, there were no significant changes in nutritional assessment variables, such as weight, BMI, and others, nor in occupational therapy assessment variables, such as FIM. A total of 24 patients transitioned from interval to endurance training before week 8, including one patient who initially required supplemental oxygen during the training program. The 6MWD improved from 483.29 ± 142.95 m at baseline to 527.79 ± 137.59 m at 8 weeks (*p* < 0.001). Significant increases were observed in lower extremity strength measured by 1'STS (number of repetitions: 25 ± 12.81 vs. 36 ± 13.42, *p*-value <0.001), upper extremity strength measured by dynamometry (28.29 ± 13.02 vs. 33.29 ± 13.95, *p*-value >0.005), and maximum dynamic inspiratory pressure measured by S-Index (*p*-value <0.005). Fifteen patients underwent motor intervention before week 8, resulting in improved Tinetti Balance and Tinetti gait scores of 14.80 ± 1.82 and 10.53 ± 2.99, respectively (*p* < 0.05, Table 1). Additionally, pain intervention was performed in 6 patients, and all measurements showed significant improvement (Table 1, *p* < 0.05).

Approximately 50% of patients were referred for psychological or cognitive therapy. Among the 13 patients who received psychological intervention, anxiety levels were significantly reduced from moderate anxiety to no anxiety or lower levels (*p*-vale <0.005). Similar improvements were observed for depression levels (Table 1). There were no significant differences between men and women in baseline levels of anxiety and depression, nor after psychological support (*p* > 0.05, Supplementary Table S4). In 56.2% of patients who underwent cognitive intervention (*n* = 18), significant improvements were observed in ability and skill scores (*p*-value <0.05), although no significant changes were found in performance on the strategy item.

For all patients who underwent rehabilitation (*n* = 32), regression models were developed to assess the change in Delta SF36 score as the outcome. The best model, determined through backward selection, comprised four shift Deltas: physical function, physical role, general health, and mental health (*p*-values >0.001, *R*^2^ = 0.907 and adjusted *R*^2^ = 0.893). Interestingly, demographic and clinical history characteristics did not emerge as predictive factors for favorable responses to rehabilitation (*n* = 15), defined as an increase of ≥30% in the Delta of change in the total score of SF36, nor for poor responders (*n* = 17). However, a logistic regression analysis revealed that sex exhibited modest significance as a predictor factor (*p* = 0.0472).

The classification models for dropout patients, utilizing the top 10 highest-ranking features across 100 iterations, achieved an average accuracy of 68.07% with a standard deviation of 0.06 and 0.45–0.86 as minimum—maximum, respectively. The most influential predictors for treatment adherence were observed at baseline and included the following variables: level of dyspnea at the end of the 6MWT, Tinetti balance score, percentage achievement in Tinetti balance, level of fatigue and dyspnea at the end of the 1'STS, vitality, and physical role scores from the SF36 questionnaire, and the presence of comorbidities such as DM, HTA, and dyslipidemia. The model exhibited approximately 65.6% true negatives (TN) and 66.7% true positives (TP), with false negatives (FN) and false positives (FP) being less than 35%.

## 4. Discussion

In Chile, as in other parts of the world, the prevalence of PICS among COVID-19 survivors is notably higher than among other patients who have experienced critical illness (6, 8). This increased prevalence can be attributed to factors such as isolation from their families and uncertainty about the prognosis (6). Furthermore, the closure of many healthcare services during the pandemic exacerbated this situation, resulting in delays in the initiation of post-hospitalization rehabilitation for COVID-19 survivors and a loss of treatment continuity for other diseases (5). COVID-19 survivors often experience lifelong repercussions, stemming from both the illness itself and their intensive care experience. Studies have demonstrated that impairments in physical, psychological, or cognitive domains are prevalent among COVID-19 UCI survivors (6). Consequently, these survivors will encounter complex physical and emotional sequelae that pose significant challenges for healthcare services (3, 5, 49).

This study, which marks the first comprehensive rehabilitation initiative in northern Chile to the authors' knowledge, involved 44 survivors of severe and moderate COVID-19. The results unveiled significant improvements after an 8-week multidisciplinary rehabilitation program across various aspects of their health, including quality of life, functionality, exercise capacity, muscular strength, mental health, and cognitive function. Particularly notable was the improvement in cardiorespiratory capacity, as measured by the 6MWT. This outcome aligns with findings from other studies that employed the 6MWT after 2–4 weeks (13) and 3 months (8) of a multidisciplinary rehabilitation program. The positive impact on exercise capacity suggests potential advancements in gait and balance among the subjects, a notion further supported by the observed improvements in the Tinetti Balance and Tinetti Gait scores. These findings are consistent with other reports where patients achieved independent walking by the end of their treatment (16). In accordance with a similar study (8), a significant improvement was observed in the muscular strength and endurance of the upper body as determined by the hand grip test. Lung function, assessed through various methods in different post-COVID-19 rehabilitation programs, including forced expiratory volume in 1 s (FEV_1_), forced vital capacity (FVC) (8, 13), and maximal inspiratory pressure (MIP) (8), consistently exhibited significant improvement, as was the case with our results for the S-Index. These consistent findings from similar studies (8, 12–14, 16), underscore the feasibility and efficacy of multidisciplinary rehabilitation programs for COVID-19 survivors (4).

### 4.1. Morbidity characteristics of severe COVID-19 patients in northern Chile

Our study examined participants from northern Chile who had experienced severe COVID-19 revealing distinct morbidity characteristics. Notably, there was a prevalence of 55.17% for hypertension and a 20.68% for diabetes mellitus among participants. Additionally, 31.03% of the participants had a history of respiratory disease. Their average length of stay in the intensive care unit (ICU) was 54.89 days, and their average body mass index (BMI) was 32.40 kg/m^2^.

These characteristics differ from other studies. For instance, a study conducted by Chadli (42) at Ibn Rochd CHU (Casablanca, Morocco) reported a lower proportion of hypertension (34.1%) and respiratory disease (4.9%), but a higher prevalence of diabetes mellitus (43.9%). Moreover, they observed a shorter average ICU stay (8.42 days) and a lower average BMI (25.34 kg/m^2^). Another study conducted in Leuven, Belgium (8), implemented a multidisciplinary respiratory rehabilitation program for 22 patients after COVID-19 hospitalization, which included group sessions over 12 weeks. In this study, only 23% of the patients had a history of pulmonary disease, and the average hospital stay was 29 days, both lower than in our study.

In contrast, our study aligns with Raman et al. (3), where individuals with severe COVID-19 cases displayed a similar average BMI of 32.40 kg/m^2^. Raman et al. (3) examined fifty-eight survivors of moderate to severe COVID-19 infection who were discharged from Oxford University Hospitals National Health Service Foundation Trust (Banbury, Oxfordshire) after 2–3 months. However, their study showed a lower prevalence of hypertension (37.9%) and diabetes mellitus (15.5%), but a higher proportion of patients with a history of respiratory disease (39.7%) compared to our results. Additionally, they had a shorter median ICU stay, lasting only 8.5 days. Notably, a common point among these studies was the higher prevalence of men among severe COVID-19 cases, which aligns with our data showing 68.96% male participants (3, 5, 8, 42).

A population more similar to our study could be expected from the research conducted by Imamura et al. (16) in São Paulo, Brazil. However, in their retrospective case series (*n* = 27) of patients who received intensive inpatient rehabilitation, they found an average BMI of 27.57 kg/m^2^, which is considerably lower than what we observed in our study. Additionally, the average length of stay in the ICU was shorter (30.04 days).

The unique morbidity characteristics of severe COVID-19 patients in northern Chile underscore the importance of regional variability in impact of the disease. Further research is warranted to comprehend the underlying factors influencing these differences in patient profiles.

### 4.2. Psychological and emotional context of post-COVID-19 survivors

Among COVID-19 survivors, it has been documented that one of the most common post-ICU referrals is to a psychologist or psychiatrist (22%) (7). The psychological and emotional context of post-COVID-19 survivors can vary depending on the population under study. For example, Chadli (42) observed that 14.6% of participants experienced moderate to severe depression, while 12.2% reported severe anxiety. In the study of Pistarini et al. (40) on cognitive and emotional disturbances caused by COVID-19 (*n* = 20) in the rehabilitation unit of San Raffaele Hospital in Milan, Italy, approximately 40% of patients exhibited symptoms of mild to moderate depression. In the Latin American study conducted by Imamura et al. (16), the percentages of moderate to severe depression and anxiety were 0% and 13.64%, respectively. In contrast, our study found higher levels of moderate to severe depression (84.61%) and moderate to severe anxiety (84.61%). These differences highlight the importance of local demographic and clinical studies, as the context of each population and country can influence individual characteristics and impact the results of a multidisciplinary rehabilitation program.

### 4.3. Discontinuity in post-COVID-19 programs

In this context, it is possible that the demographic and clinical characteristics of our population may explain some of our results. Non-continuity in post-COVID rehabilitation programs has been observed before (16), where the main reason was voluntary discharge. In our study, twelve patients discontinued the program before 8 weeks for various reasons, including repeated absences, lack of motivation, loss of telephone follow-up, among others. Explaining this phenomenon, which has multiple facets and occurs in the context of a pandemic, is complex. One possible explanation could be associated with our population of obese survivors, who were likely obese before their COVID-19 infection. Obesity was one of the most common comorbidities observed in severe cases of SARS-CoV-2 (8, 50), and it has been linked to poor attendance rates and compliance, hindering treatment effectiveness. The presence of barriers to behavior change, such as poor motivation, lack of time, health and physical limitations, negative thoughts/moods, socioeconomic factors, lack of enjoyment of exercise, and other determinants of adherence, is a characteristic of this disease. On the other hand, the high prevalence of moderate to severe depression and anxiety among our participants is often accompanied by symptoms commonly observed in the post-illness stage, such as frequent recall of traumatic memories, insomnia, and emotional lability (40, 51). These symptoms have been associated with adherence issues and persistence in therapies (52).

### 4.4. Predicting dropout individuals: a proposed classification model

One possible solution to address the issue of patients dropping out during rehabilitation programs is to implement an initial screening to identify individuals with a higher likelihood of discontinuing their participation. Such an approach could help saving both human and structural resources while also enabling the offer of more suitable treatment alternatives tailored to individual preferences and contexts. In line with this strategy, our study introduces a preliminary classification model designed to predict individuals at risk of dropping out. This model incorporates the most influential predictors for treatment adherence, all of which were assessed at baseline.

These predictive variables encompass factors such as the level of dyspnea at the end of the 6MWT, Tinetti balance score, percentage achievement in Tinetti balance, levels of fatigue and dyspnea at the end of the 1'STS, vitality and physical role scores from the SF36 questionnaire, as well as the presence of comorbidities such as DM, HTA, and dyslipidemia. It is noteworthy that these characteristics align with observations from various studies involving post-COVID patients across different geographical locations, where the presence of comorbidities like HT and, DM has also been noted (3, 8, 17, 42).

Additionally, our findings suggest a linear relationship between the duration of rehabilitation and the physical (muscular strength, exercise capacity) and functional improvements (gait, balance, daily activities, among others) achieved by the end of the rehabilitation program (16). On the other hand, the research by Imamura et al. (16) indicated that baseline psychological and cognitive functions at admission did not significantly influence the duration of rehabilitation interventions or the functional outcomes achieved upon discharge. Consequently, these variables were not considered as predictors in our dropout model.

An important aspect of our proposed model is its reliance on low-cost, validated tools that can be readily employed in most healthcare settings. These tools include the 6MWT, 1'STS, SF36 questionnaire, and a comorbidity interview, making the model both practical and accessible for widespread implementation.

### 4.5. Factors influencing therapeutic focus

Furthermore, our regression model aimed at predicting changes in the SF-36 total score was influenced by four key components of quality of life: physical function, physical role, general health, and mental health. In a study conducted by Líška et al. (53) where the SF-36 questionnaire was administered to long-COVID patients (*n* = 469), the following components showed mean scores of 66.2 ± 25.4 in physical function, 34.1 ± 21.4 in physical role, 35.8 ± 16.1 in general health, and 38.6 ± 16.0 in mental health. When compared to a control group, all these components showed significant differences. In our patient sample, at baseline conditions, we observed a lower mean score for physical function (59.6 ± 19.7), while higher mean scores were reported for physical role, general health, and mental health (39.0 ± 23.4, 58.8 ± 19.7, 58.8 ± 118.2, respectively). This suggests an opportunity to focus therapeutic efforts on these specific components, potentially reducing costs associated with therapies targeting other aspects of quality of life of COVID-19 survivors.

In our study, demographic and clinical history characteristics were not predictive of whether individuals would be good or poor responders to the multidisciplinary rehabilitation program. This may be due to therapy response being influenced by factors such as sociocultural and socioeconomic variables, which were not considered into account in this study but should be considered in the rehabilitation of COVID-19 survivors (5). It has been observed that the presence of risk factors, such as living in overcrowded conditions, precarious housing, lack of accessible medical care, and employment in higher-risk environments (54), can influence the therapy response of more vulnerable patients, including those within the public healthcare system. While a more comprehensive sociodemographic study of the population in northern Chile who survived COVID-19 is needed, these preliminary findings provide initial insights into population behavior and factors to consider in a multidisciplinary rehabilitation program.

Despite the notable improvements in functional outcomes that underscore the potential of a comprehensive multidisciplinary rehabilitation program, this prospective study does have limitations. These, include a relatively small sample size and the absence of a control group. Additionally, we acknowledge the differences in group sizes, as therapy was customized to meet the individual needs of COVID-19 survivors based on recommendations. However, it is worth to note that this study aims to document the experiences of a rehabilitation service during a pandemic period when there was limited scientific evidence from randomized controlled trials. This is particularly significant in the context of patients reliant on the public healthcare system in Chile or with limited resources, highlighting the relevance of this manuscript for developing countries.

## 5. Conclusion

This study demonstrates significant improvements in quality of life, irrespective of the type of therapy received, accompanied by positive changes in functional performance and reductions in mental and cognitive burdens within an 8-week rehabilitation program. Additionally, the successful identification of patients at risk of dropout using cost-effective, outpatient, and clinically applicable test offers a practical solution for optimizing human and structural resources in low-resource clinical settings. These valuable insights will serve as a guide for the more effective development of PICS rehabilitation programs, ultimately benefiting COVID-19 survivors and enhancing overall healthcare outcomes in our region. Further research and the implementation of these findings hold the potential to improve patient outcomes and resource allocation in the future.

## Data Availability

The raw data supporting the conclusions of this article will be made available by the authors, without undue reservation.
