# Supplementary material for: Quality of life, exercise capacity, cognition, and mental health of Chilean patients after COVID-19: an experience of a multidisciplinary rehabilitation program at a physical and rehabilitation medicine unit
Source: Front Rehabil Sci. 2023 Dec 1;4:1274180. doi: 10.3389/fresc.2023.1274180 (PMC10722286; doi:10.3389/fresc.2023.1274180)
Supplement: Supplementary file 1 [file Datasheet1.pdf]

Supplementary Material

Figure S1. The standardized guideline of exercises of muscular strength

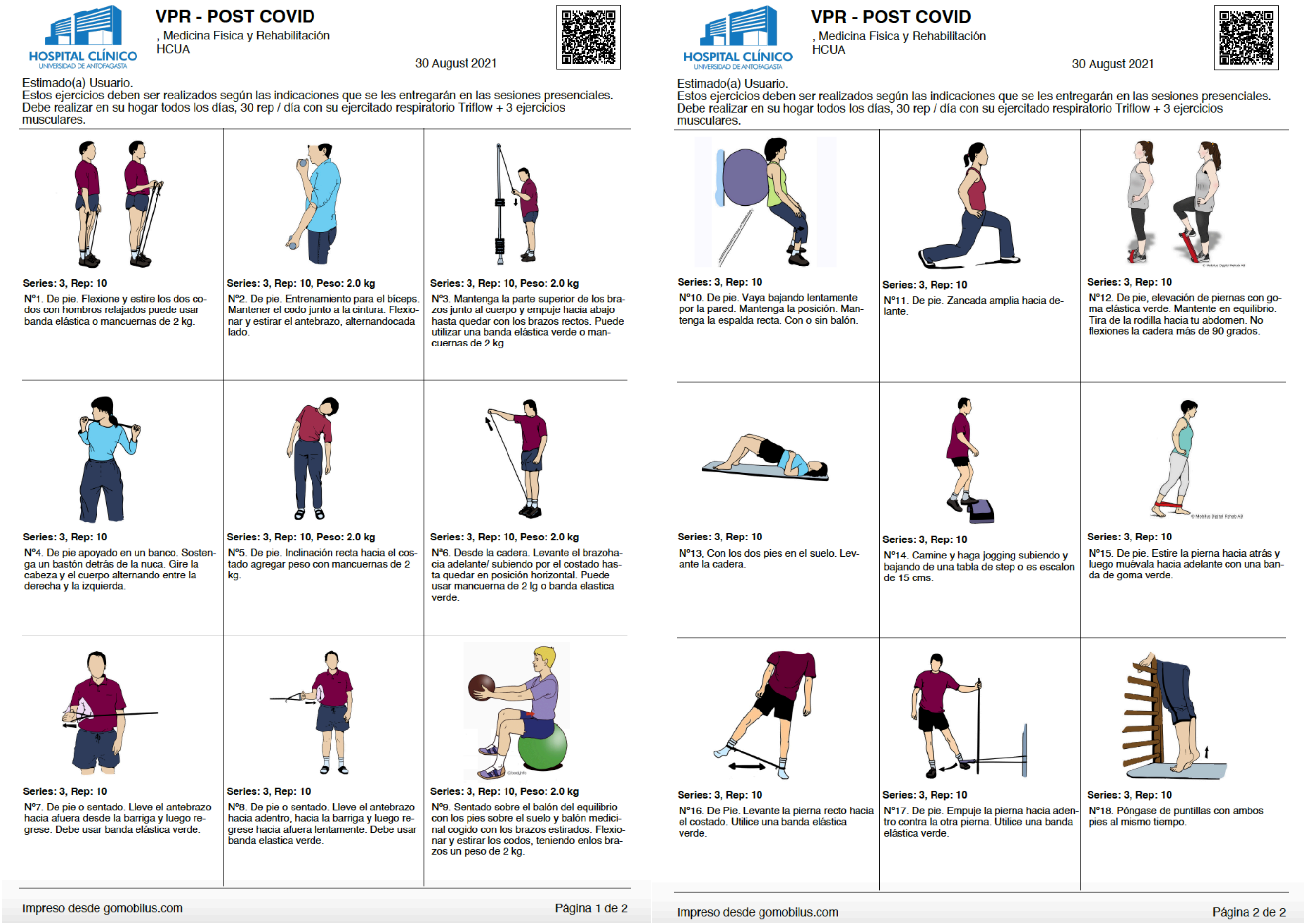

HOSPITAL CLÍNICO
UNIVERSIDAD DE ANTOFAGASTA

VPR - POST COVID
, Medicina Fisica y Rehabilitación
HCUA

30 August 2021

Estimado(a) Usuario.
Estos ejercicios deben ser realizados según las indicaciones que se les entregarán en las sesiones presenciales.
Debe realizar en su hogar todos los días, 30 rep / día con su ejercitado respiratorio Triflow + 3 ejercicios musculares.

Series: 3, Rep: 10
Nº1. De pie. Flexione y estire los dos codos con hombros relajados puede usar banda elástica o mancuernas de 2 kg.

Series: 3, Rep: 10, Peso: 2.0 kg
Nº2. De pie. Entrenamiento para el bíceps. Mantener el codo junto a la cintura. Flexionar y estirar el antebrazo, alternandocada lado.

Series: 3, Rep: 10, Peso: 2.0 kg
Nº3. Mantenga la parte superior de los brazos junto al cuerpo y empuje hacia abajo hasta quedar con los brazos rectos. Puede utilizar una banda elástica verde o mancuernas de 2 kg.

Series: 3, Rep: 10
Nº4. De pie apoyado en un banco. Sostenga un bastón detrás de la nuca. Gire la cabeza y el cuerpo alternando entre la derecha y la izquierda.

Series: 3, Rep: 10, Peso: 2.0 kg
Nº5. De pie. Inclinación recta hacia el costado agregar peso con mancuernas de 2 kg.

Series: 3, Rep: 10, Peso: 2.0 kg
Nª6. Desde la cadera. Levante el brazohacia adelante/ subiendo por el costado hasta quedar en posición horizontal. Puede usar mancuerna de 2 lg o banda elastica verde.

Series: 3, Rep: 10
Nº7. De pie o sentado. Lleve el antebrazo hacia afuera desde la barriga y luego regrese. Debe usar banda elástica verde.

Series: 3, Rep: 10
Nº8. De pie o sentado. Lleve el antebrazo hacia adentro, hacia la barriga y luego regrese hacia afuera lentamente. Debe usar banda elastica verde.

Series: 3, Rep: 10, Peso: 2.0 kg
Nº9. Sentado sobre el balón del equilibrio con los pies sobre el suelo y balón medicinal cogido con los brazos estirados. Flexionar y estirar los codos, teniendo enlos brazos un peso de 2 kg.

Impreso desde gomobilus.com   Página 1 de 2

HOSPITAL CLÍNICO
UNIVERSIDAD DE ANTOFAGASTA

VPR - POST COVID
, Medicina Fisica y Rehabilitación
HCUA

30 August 2021

Estimado(a) Usuario.
Estos ejercicios deben ser realizados según las indicaciones que se les entregarán en las sesiones presenciales.
Debe realizar en su hogar todos los días, 30 rep / día con su ejercitado respiratorio Triflow + 3 ejercicios musculares.

Series: 3, Rep: 10
Nº10. De pie. Vaya bajando lentamente por la pared. Mantenga la posición. Mantenga la espalda recta. Con o sin balón.

Series: 3, Rep: 10
Nº11. De pie. Zancada amplia hacia delante.

Series: 3, Rep: 10
Nº12. De pie, elevación de piernas con goma elástica verde. Mantente en equilibrio. Tira de la rodilla hacia tu abdomen. No flexiones la cadera más de 90 grados.

Series: 3, Rep: 10
Nº13, Con los dos pies en el suelo. Levante la cadera.

Series: 3, Rep: 10
Nº14. Camine y haga jogging subiendo y bajando de una tabla de step o es escalon de 15 cms.

Series: 3, Rep: 10
Nº15. De pie. Estire la pierna hacia atrás y luego muévala hacia adelante con una banda de goma verde.

Series: 3, Rep: 10
Nº16. De Pie. Levante la pierna recto hacia el costado. Utilice una banda elástica verde.

Series: 3, Rep: 10
Nº17. De pie. Empuje la pierna hacia adentro contra la otra pierna. Utilice una banda elástica verde.

Series: 3, Rep: 10
Nº18. Póngase de puntillas con ambos pies al mismo tiempo.

Impreso desde gomobilus.com   Página 2 de 2

Figure S2. The standardized guideline of exercises of motor protocol

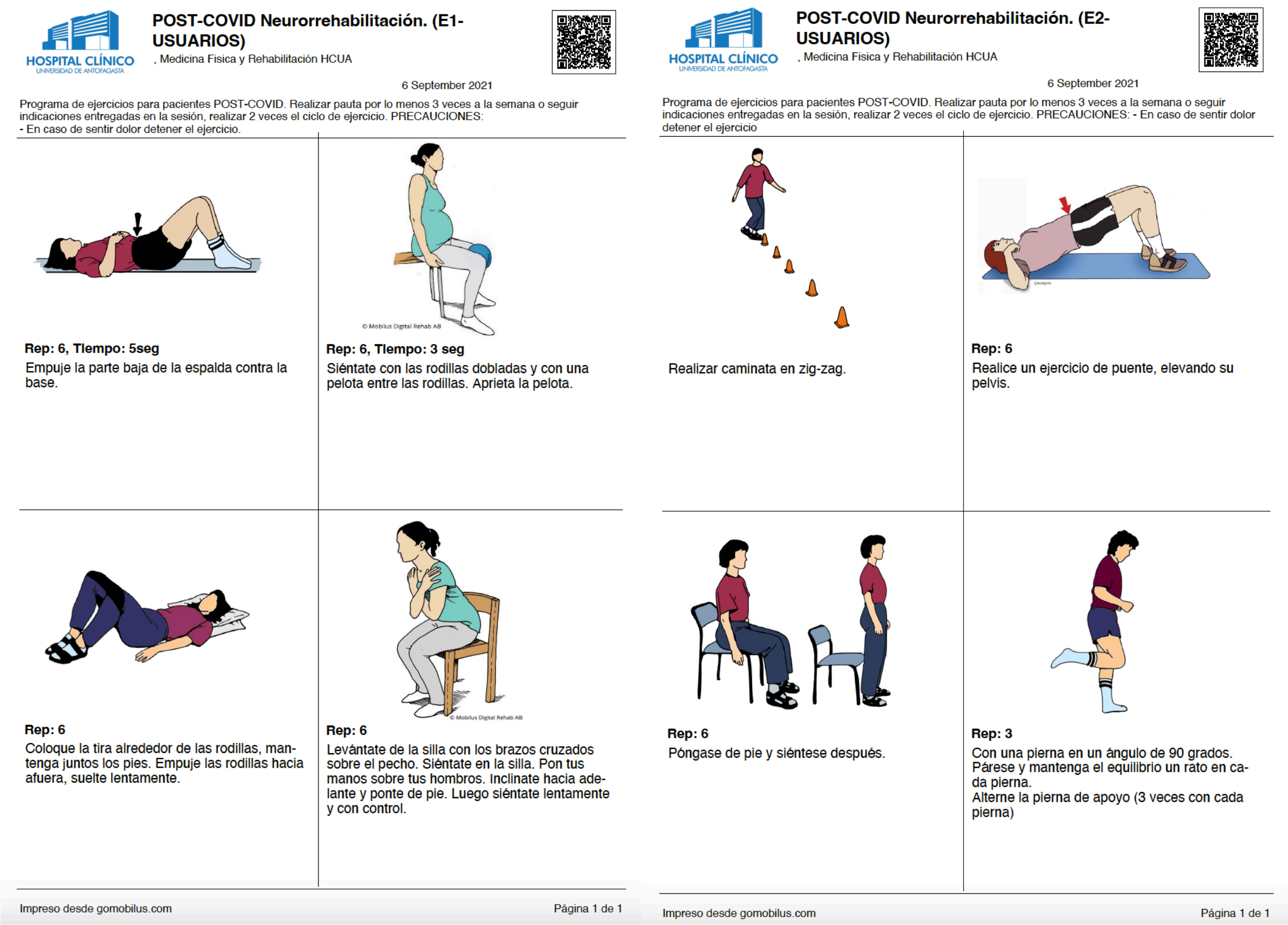

HOSPITAL CLÍNICO
UNIVERSIDAD DE ANTOFAGASTA

POST-COVID Neurorrehabilitación. (E1-USUARIOS)
, Medicina Fisica y Rehabilitación HCUA

6 September 2021

Programa de ejercicios para pacientes POST-COVID. Realizar pauta por lo menos 3 veces a la semana o seguir indicaciones entregadas en la sesión, realizar 2 veces el ciclo de ejercicio. PRECAUCIONES:
- En caso de sentir dolor detener el ejercicio.

Rep: 6, Tiempo: 5seg
Empuje la parte baja de la espalda contra la base.

© Mobilus Digital Rehab AB
Rep: 6, Tiempo: 3 seg
Siéntate con las rodillas dobladas y con una pelota entre las rodillas. Aprieta la pelota.

Rep: 6
Coloque la tira alrededor de las rodillas, mantenga juntos los pies. Empuje las rodillas hacia afuera, suelte lentamente.

© Mobilus Digital Rehab AB
Rep: 6
Levántate de la silla con los brazos cruzados sobre el pecho. Siéntate en la silla. Pon tus manos sobre tus hombros. Inclínate hacia adelante y ponte de pie. Luego siéntate lentamente y con control.

Impreso desde gomobilus.com — Página 1 de 1

HOSPITAL CLÍNICO
UNIVERSIDAD DE ANTOFAGASTA

POST-COVID Neurorrehabilitación. (E2-USUARIOS)
, Medicina Fisica y Rehabilitación HCUA

6 September 2021

Programa de ejercicios para pacientes POST-COVID. Realizar pauta por lo menos 3 veces a la semana o seguir indicaciones entregadas en la sesión, realizar 2 veces el ciclo de ejercicio. PRECAUCIONES: - En caso de sentir dolor detener el ejercicio

Realizar caminata en zig-zag.

Rep: 6
Realice un ejercicio de puente, elevando su pelvis.

Rep: 6
Póngase de pie y siéntese después.

Rep: 3
Con una pierna en un ángulo de 90 grados. Párese y mantenga el equilibrio un rato en cada pierna.
Alterne la pierna de apoyo (3 veces con cada pierna)

Impreso desde gomobilus.com — Página 1 de 1

POST-COVID Neurorrehabilitación.(E3-USUARIOS)

HOSPITAL CLÍNICO
UNIVERSIDAD DE ANTOFAGASTA

, Medicina Fisica y Rehabilitación HCUA

6 September 2021

Programa de ejercicios para pacientes POST-COVID. Realizar pauta por lo menos 3 veces a la semana o seguir indicaciones entregadas en la sesión, realizar 2 veces el ciclo de ejercicio. PRECAUCIONES:
- En caso de sentir dolor detener el ejercicio

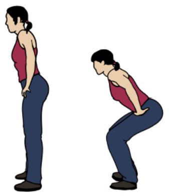

Rep: 6

Espalda recta, dóblese en la cadera, el peso sobre los talones. Siéntese.

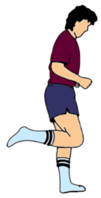

Rep: 3

Con una pierna en un ángulo de 90 grados. Párese y mantenga el equilibrio un rato en cada pierna. Alterne la extremidad que se encuentra en apoyo.
Realice 3 repeticiones con cada extremidad.

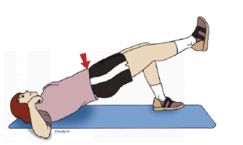

Rep: 6

Realice un puente mientras estira una de sus piernas.
Realice 3 repeticiones con cada extremidad.

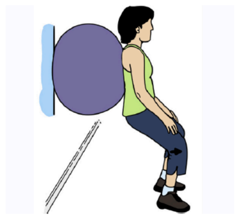

Rep: 6

IMPORTANTE: No utilice balon.
Vaya bajando lentamente por la pared. Mantenga la posición. Mantenga la espalda recta.

Figure S3. The standardized guideline of exercises of pain protocol

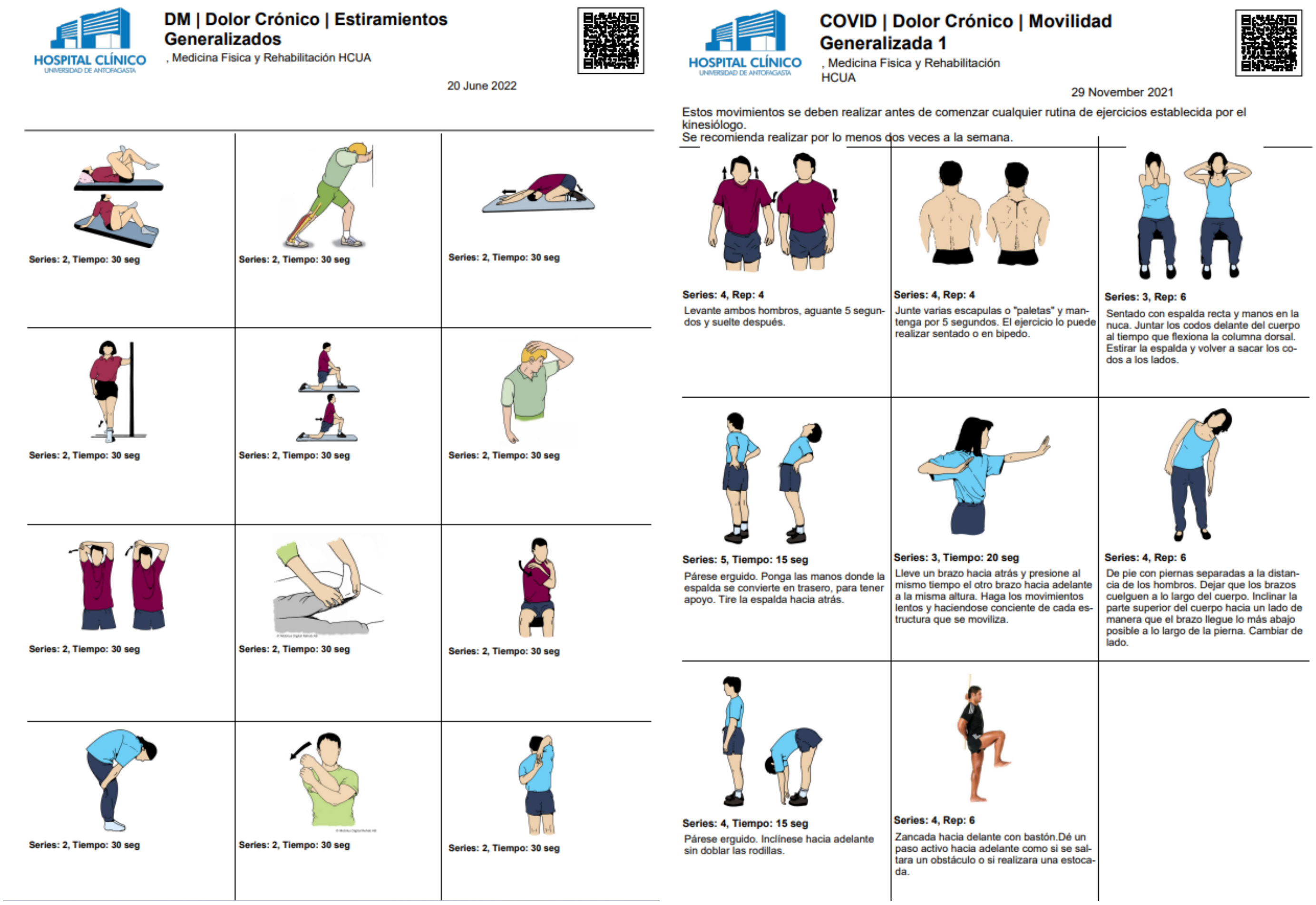

HOSPITAL CLÍNICO
UNIVERSIDAD DE ANTOFAGASTA

DM | Dolor Crónico | Estiramientos Generalizados
, Medicina Fisica y Rehabilitación HCUA

20 June 2022

Series: 2, Tiempo: 30 seg

Series: 2, Tiempo: 30 seg

Series: 2, Tiempo: 30 seg

Series: 2, Tiempo: 30 seg

Series: 2, Tiempo: 30 seg

Series: 2, Tiempo: 30 seg

Series: 2, Tiempo: 30 seg

Series: 2, Tiempo: 30 seg

Series: 2, Tiempo: 30 seg

Series: 2, Tiempo: 30 seg

Series: 2, Tiempo: 30 seg

Series: 2, Tiempo: 30 seg

HOSPITAL CLÍNICO
UNIVERSIDAD DE ANTOFAGASTA

COVID | Dolor Crónico | Movilidad Generalizada 1
, Medicina Fisica y Rehabilitación HCUA

29 November 2021

Estos movimientos se deben realizar antes de comenzar cualquier rutina de ejercicios establecida por el kinesiólogo.
Se recomienda realizar por lo menos dos veces a la semana.

Series: 4, Rep: 4
Levante ambos hombros, aguante 5 segundos y suelte después.

Series: 4, Rep: 4
Junte varias escapulas o "paletas" y mantenga por 5 segundos. El ejercicio lo puede realizar sentado o en bipedo.

Series: 3, Rep: 6
Sentado con espalda recta y manos en la nuca. Juntar los codos delante del cuerpo al tiempo que flexiona la columna dorsal. Estirar la espalda y volver a sacar los codos a los lados.

Series: 5, Tiempo: 15 seg
Párese erguido. Ponga las manos donde la espalda se convierte en trasero, para tener apoyo. Tire la espalda hacia atrás.

Series: 3, Tiempo: 20 seg
Lleve un brazo hacia atrás y presione al mismo tiempo el otro brazo hacia adelante a la misma altura. Haga los movimientos lentos y haciendose conciente de cada estructura que se moviliza.

Series: 4, Rep: 6
De pie con piernas separadas a la distancia de los hombros. Dejar que los brazos cuelguen a lo largo del cuerpo. Inclinar la parte superior del cuerpo hacia un lado de manera que el brazo llegue lo más abajo posible a lo largo de la pierna. Cambiar de lado.

Series: 4, Tiempo: 15 seg
Párese erguido. Inclínese hacia adelante sin doblar las rodillas.

Series: 4, Rep: 6
Zancada hacia delante con bastón.Dé un paso activo hacia adelante como si se saltara un obstáculo o si realizara una estocada.

COVID | Dolor Crónico | Ejercicios de Respiratorio

, Medicina Fisica y Rehabilitación HCUA

7 September 2021

Aprender a respirar es primordial para manejar nuestras dolencias. ¿Te parece si comenzamos con estos simples ejercicios?
Te sugiero que lo realicemos 1 ves al día y/o cada vez que la ansiedad se este ganando el protagonismo durante tu día.

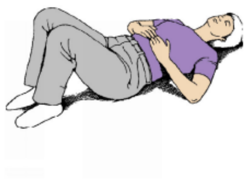

Series: 10

Reliza respiraciones profundas. Donde al tomar aire (inspiración) deberás inflar tu abdomen y al botar el aire (espiración) deberás entrar es abdomen.
10 ciclos respiratorios
1 Ciclo respiratorio = a Insipiración y espiración

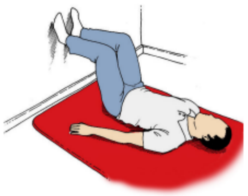

Series: 10

Acuéstese boca arriba con los pies contra la pared, 90 grados en la cadera. Pise alternativamente, desde el centro. Integre la respiración gradualmente. 10 ciclos respiratorios

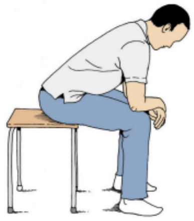

Series: 5

Tire de los hombros hacia abajo / tire de los omóplatos hacia atrás y presione con los codos. Tire de la barbilla, extienda el cuello y toda la espalda desde las patas del asiento hacia arriba. Agregue el sonido "mmm..." a la espiración. Realizar 5 ciclos respiratorios

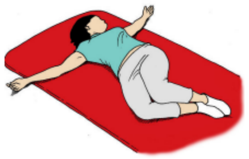

Series: 10

Acuéstese con las piernas dobladas y la nariz apuntando hacia el techo. Las piernas caen suavemente hacia un lado y la cabeza hacia el otro. Tire de las piernas hacia arriba a la posición inicial. Continuar en la otra dirección. Integrar movimiento, respiración y sonidos "m". Las piernas caen durante la inspiración, y al espirar las levanta. Toma la potencia del centro y del sonido "m".

COVID | Dolor Crónico | Neurodinamia EESS

, Medicina Fisica y Rehabilitación HCUA

7 September 2021

Estos ejercicios ayudarán a restaurar una homeostasis alterada en el sistema neuro-inmune mediante la movilización del sistema nervioso y otras estructuras que lo rodean. A través de ella podemos tratar los mecanismos de hipersensibilidad del tejido neural. Realice los ejercicios por lo menos 1 vez al día.

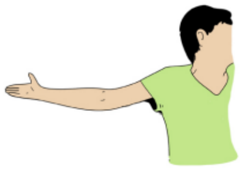

Series: 5, Tiempo: 30 seg

Deslizamiento del nervio ulnar, tumbado en la cama sin cojín con brazo extendido y pulgar hacia abajo contra la colchoneta, al tiempo que la cabeza se inclina en ángulo hacia el otro lado. Volver lentamente. 5 x 30 segundos con cada brazo mañana y tarde.

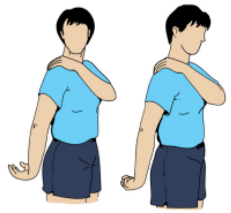

Series: 5, Tiempo: 30 seg

Imite la posición de la imagen y con el otro brazo trate de descender el hombro del mismo lado. Mantenga la posición 30 seg y repitala 3 veces con cada brazo.

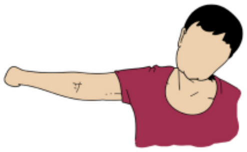

Series: 5

Deslizamiento del nervio radial. Desplazar el brazo con muñeca flexionada y dedos entrelazados como en la figura y con la cabeza inclinada en dirección opuesta.

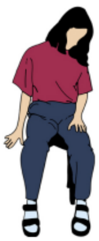

Series: 5, Tiempo: 30 seg

Deje que baje el hombro. Incline la cabeza y el cuerpo hacia la cadera opuesta hasta notar tirantez. Abra las manos en forma de abanico.

HOSPITAL CLÍNICO
UNIVERSIDAD DE ANTOFAGASTA

COVID | Dolor Crónico | Neurodinamia EEII

, Medicina Fisica y Rehabilitación HCUA

7 September 2021

Restaurar una homeostasis alterada en el sistema neuro-inmune mediante la movilización del sistema nervioso y otras estructuras que lo rodean. A través de ella podemos tratar los mecanismos de hipersensibilidad del tejido neural que favorecen las dolencias física por irritación de tejido. Realiza estos ejercicios 1 vez a la semana.

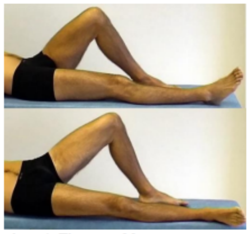

Series: 4, Tiempo: 30 seg

Acostado boca arriba. Con una pierna flexiionada y la otra extendida. Realiza un movimiento de rotación externa de cadera y manten la posición durante 30 segundos y cambia de pie.

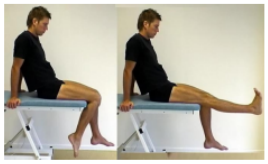

Series: 4, Tiempo: 30 seg

Sentado estira una pierna y trata de apuntar con la punte del pie la punta de la nariz. Mantiene la posición durante 30 segundos.

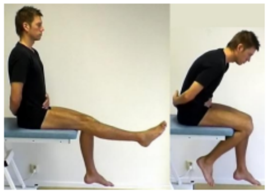

Series: 4, Tiempo: 20 seg

Sentado, estira una pierna al mismo tiempo que inclinamos en tronco y flexionamos el cuello. Posterior a eso realiza el mismo movimiento de manera inversa. Manten 20 segundos en cada posición.

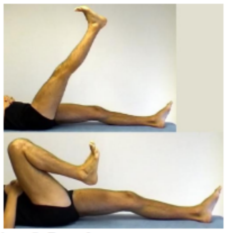

Series: 5, Rep: 3

Acostado, estira una pierna y la otra tambien extendida realiza una flexión de la cadera para terminar con una flexión de rodilla.
Realiza el movimiento constantemente de manera lenta con cada pierna.

Figure S4. The standardized guideline of exercises of occupational therapy protocol

Programa Suplementario Terapia Ocupacional Extremidad Superior Post-COVID-19

15 argollas / 3 series

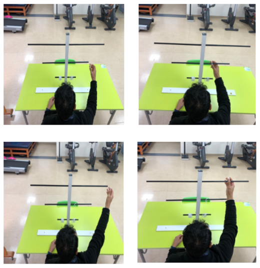

1. Prensión pentadigital con flexión de hombro progresiva.

Siéntese erguido frente a una mesa. Ubica una estructura de 3 niveles. Usando su brazo débil, inserte las argollas en cada una de las en el nivel más bajo. Una vez que las haya insertado todas, continúe con el nivel medio y luego alto. Intente mantener el cuerpo quieto durante el ejercicio y evite compensar el movimiento con su hombro.

10 series / 3 repeticiones

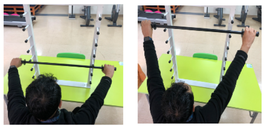

2. Flexión de hombro contra gravedad con prensión palmar cilíndrica.

Siéntese erguido frente a una mesa con ambos pies posicionados en el suelo. Ubica una estructura de 7 niveles. Usando ambos brazos, toma una barra desde los extremos utilizando el cierre completo de tu mano. Posiociona la barra en el primer nivel y continúa subiendo al siguiente de uno en uno hasta la máxima altura alcanzada. Una vez logrado, baja deteniéndote en cada nivel de la estrucutra.

10 repeticiones/ 3 series

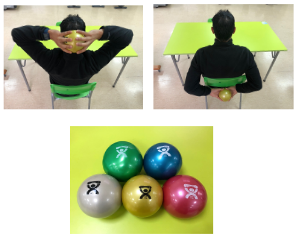

3. Flexión, Abducción, Aducción, Rotación Externa y Rotación Interna de hombro, con agarre palmar esférico.

Siéntese erguido sobre una silla. Posiciones ambos pies de forma paralela en el suelo. Tome un balón de 0.5 kilos con su mano débil. Trasládelo por detrás de la cabeza y realice el intercambio a su mano no afectada. Por delante de su tronco, realice el traslado nuevamente hacia su mano afectada y diríjalo a la zona baja de su espalda. Ttraslade el balón hacia su mano no afectada y continúe el ciclo. Incremente el peso del balón a medida que presente mayor fuerza.

10 argollas / 3 series

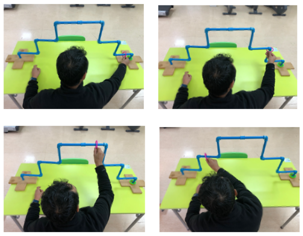

4. Rotación de tronco y precisión del movimiento.

Siéntese erguido frente a una mesa. Ubica una estructura con forma de escalera. Posicione las argollas en el lado del hemicuerpo afectado. Traslade cada una de las argollas al otro extremo del circuito sin tocar la estrucutra. Intente mantener el tronco erguido durante el ejercicio, permitiendo la rotación del tronco cuando realice cruce de línea media. Evite realizar lateralizaciones del cuerpo.

10 conos/ 3 repeticiones

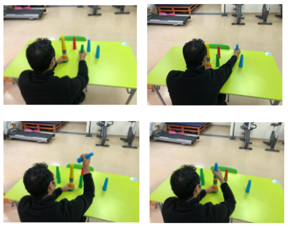

5. Integración bilateral con agarre cilíndrico contra gravedad.

Siéntese erguido frente a una mesa. Ubique 10 conos a diversas distancias de usted. Con su hemicuerpo afectado, alcance cada uno de los conos. Inserte los conos unos sobre otros de manera vertical, mientras los sostiene con su mano no afectada.

30 repeticiones/ 3 series

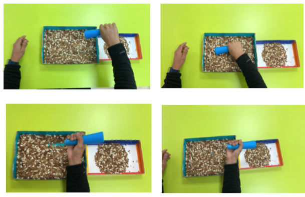

6. Pronosupinación de antebrazo.

Siéntese erguido frente a una mesa. Ubique una fuente baja sobre usted con semillas. En posición prona del antebrazo afectado, tome el cono y llénelo de semillas. Traslade las semillas a otro recipiente con el antebrazo en posición supina. Mientras realice esta tarea, mantenga todos sus dedos en contacto con el cono. Evite realizar movimientos compensatorios con

15 repeticiones/ 3 series

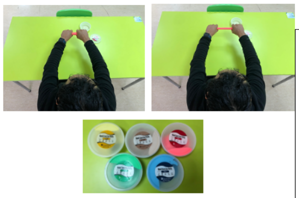

7. Fuerza agarre palmar completo.

Siéntese erguido frente a una mesa. Tome la masilla terapéutica nivel soft con ambas manos. Extienda la masilla sin apoyo de sus antebrazos en la mesa. Mantenga una distancia equitativa entre su hemicuerpo afectado y no afectado. Evite rotar la masa y realizar movimientos compensatorios con sus hombros. Incremente la densidad de la masa a medida que adquiera mayor fuerza.

15 repeticiones / 3 series / 3 variaciones

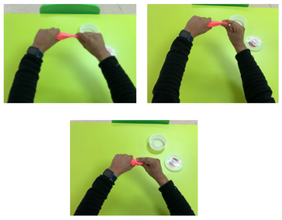

8. Fuerza prensión subterminal.

Siéntese erguido frente a una mesa. Tome la masilla terapéutica nivel soft de un extremo con su mano indemne. Con su mano débil, tome la masilla del otro extremo y extiéndala utilizando su dedo índice y pulgar. Vuelva a juntar la masa y realice la misma acción con su dedo medio y pulgar. Para finalizar, realice la misma acción utilizando los dedos índice y medio en oposición al pulgar. Extienda la masilla sin apoyo de sus antebrazos en la mesa. Mantenga una distancia equitativa entre su hemicuerpo afectado y no afectado. Evite rotar la masa y realizar movimientos comprensatorios con sus hombros. Incremente la densidad de la masa a medida que adquiera mayor fuerza.

25 pinzas/ 2 repeticiones

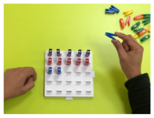
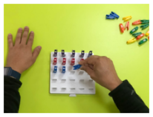
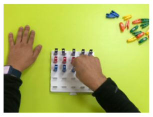

9. Coordinación motora fina con pinza subterminal.

Siéntese erguido frente a una mesa. Posicione el tablero para insertar las pinzas de ropa frente a usted. Con su mano débil, tome una pinza de ropa utilizando los dedos índice y pulgar. Insértela en la superficie correspondiente. Una vez haya completado el tablero, retire todas las pinzas una en una. Evite utilizar su dedo medio como medida

10 cilindros / 3 repeticiones

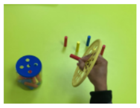
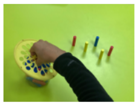

10. Fuerza de agarre en oposición penta-digital.

Siéntese erguido frente a una mesa. Posicione su mano afectada en posición de garra. Con su mano indemnse inserte la banda elástica de mano nivel dos en su mano débil. Cada dedo debe posicionarse en un orificio diferente, manteniendo la mano en garra. Tome un cilindro manteniendo todos los dedos en contacto con este e inserte en la ranura circular correspondiente. Mantenga su muñeca en posición neutra ante la flexo extensión. Evite realizar movimientos compensatorios con su hombro.

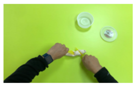
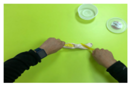
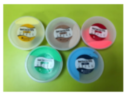

11. Coordinación bimanual en tarea de alimentación utilizando tenedor y cuchillo.

Prepare un plato de comida apto para cortar o sobre una mesa, un cuchillo y un tenedor.
Este ejercicio también puede realizarse utilizando masilla terapéutica o plasticina en lugar de alimentos. Sujeta el cuchillo con tu mano afectada y el tenedor con tu mano indemne. La forma más funcional de sujetar un cuchillo y un tenedor es teniendo el dedo índice apuntando hacia abajo del cubierto, mientras los otros dedos se enroscan alrededor del mango. Utiliza el tenedor para mantener la comida en su sitio mientras usas el cuchillo para cortar la comida con un movimiento hacia delante y hacia atrás. Si es necesario, también se puede utilizar un tapete antideslizante debajo del plato para mantenerlo en en su sitio. Incrementa la densidad de la masa hasta alcanzar una similar a la carne.

Table S1. Analysis for quality of live (questionary SF36)

Variable	Distribution	p-value (pre VS post), alpha = 0.05	d-Cohen
PRE physical function	Normal	2.015×10^{-5}	-0.9920
POST physical function	No normal		
PRE physical role	No normal	4.906×10^{-5}	-1.0034
POST physical role	Normal		
PRE body pain	Normal	2.416×10^{-4}	-0.7333
POST body pain	Normal		
PRE general health	Normal	0.005	-0.5345
POST general health	Normal		
PRE vitality	Normal	1.409×10^{-5}	-0.9099
POST vitality	Normal		
PRE social role	Normal	0.003	-0.5654
POST social role	No normal		
PRE emotional role	Normal	1.086×10^{-4}	-0.9240
POST emotional role	No normal		
PRE mental health	Normal	1.779×10^{-4}	-0.7440
POST mental health	No normal		
PRE health transition	No normal	2.635×10^{-4}	-0.8398
POST health transition	No normal		
PRE score total	Normal	8.209×10^{-8}	-1.2308
POST score total	Normal		

Table S2. Analysis for Dysfunctionality by PCFS

Variable	Distribution	p-value (pre VS post), alpha = 0.05	d-Cohen
PRE PCFS	No normal	8.223×10^{-4}	0.6891
POST PCFS	No normal		

Table S3. Description of the groups according to the type of therapy received (n=32)

**Group**	**Therapy**	**n**
1	PT + N + 0T	1
2	PT + N + P	2
3	PT + N + P + ST	7
4	PT + N + ST	5
5	PT + N + SP + OT	1
6	PT + N + P + ST + OT	1
7	PT + N	8
8	PT	1
9	N + ST	1
10	PT + P + ST	2
11	PT + OT	1
12	P	1
13	PT + ST	1

Physical therapy = PT, Nutrition = N, Occupational therapy = OT, Psychology = P, Speech therapy = ST

Table S4. Analysis for sex for anxiety level and depression level post 8-week of psychological support.

		**8-week**	**p-value X^2-test**
HAMA, anxiety level >=2	Women (n=7)	1/7(14.2%)	0.7934
	Men (n=6)	1/4 (25%)	

Anxiety levels (HAMA): 0=no anxiety, 1=mild anxiety, 2=moderate anxiety, 3=severe anxiety.

		**8-week**	**p-value X^2-test**
HAMD, depression level >=2	Women (n=7)	0/7(0%)	0.2165
	Men (n=6)	1/4 (25%)	

Depression level (HAMD): 0=not depressed, 1=mild depression, 2=moderate depression, 3=severe depression, 4=very severe depression.
